# Associations Between Comorbidity and Depressive Symptoms During COVID-19: Variation by Social Isolation?

**DOI:** 10.1093/geroni/igab046.144

**Published:** 2021-12-17

**Authors:** Jianjia Cheng, Lindsay Kobayashi

**Affiliations:** 1 School of Public Health, Ann Arbor, Michigan, United States; 2 University of Michigan, Ann Arbor, Michigan, United States

## Abstract

Adults with comorbidities are at high COVID-19 risk and may experience elevated depressive symptoms during the pandemic. We aimed to investigate the associations between comorbidity at pandemic onset and subsequent depressive symptoms and whether social isolation modified this association. Data were from monthly online questionnaires in the COVID-19 Coping Study of US adults aged ≥55 from April/May-September/October 2020 (n=4,383). Depressive symptoms were measured by the 8-item CES-D, and social isolation as “high” vs. “low” based on contact with family, friends, social organizations, and living alone. In multivariable mixed-effects models, comorbidity (≥2 vs. <2 chronic conditions) was associated with greater depressive symptoms at baseline (β=0.50; 95% CI: 0.36-0.64), this association varied negligibly by social isolation. Differences in depressive symptoms by comorbidity status at pandemic onset were consistent over the six-month follow-up. This study indicates that middle-aged and older US adults with comorbidities experienced persistently elevated depressive symptoms during the COVID-19 pandemic.

